# Phytochemical Composition, Antifungal and Antioxidant Activity of* Duguetia furfuracea* A. St.-Hill

**DOI:** 10.1155/2016/7821051

**Published:** 2016-04-05

**Authors:** Francisca Valéria Soares de Araújo Pinho, Litiele Cezar da Cruz, Nathane Rosa Rodrigues, Emily Pansera Waczuk, Celestina Elba Sobral Souza, Henrique Douglas Melo Coutinho, José Galberto Martins da Costa, Margareth Linde Athayde, Aline Augusti Boligon, Jeferson Luis Franco, Thaís Posser, Irwin Rose Alencar de Menezes

**Affiliations:** ^1^Department of Biological Chemistry, Laboratory of Pharmacology and Molecular Chemistry (LFQM), Regional University of Cariri (URCA), 63100-000 Crato, CE, Brazil; ^2^Department of Biochemistry and Molecular Biology, Postgraduate Program in Toxicological Biochemistry, Federal University of Santa Maria (UFSM), 97105-900 Santa Maria, RS, Brazil; ^3^Interdisciplinary Center for Biotechnology Research (CIPBIOTEC), Federal University of Pampa (UNIPAMPA), 97300-000 Sao Gabriel, RS, Brazil; ^4^Department of Biological Chemistry, Laboratory of Microbiology and Molecular Chemistry (LMBM), Regional University of Cariri (URCA), 63100-000 Crato, CE, Brazil; ^5^Department of Biological Chemistry, Laboratory of Studies on Natural Products (LPPN), Regional University of Cariri (URCA), 63105-000 Crato, CE, Brazil; ^6^Department of Industrial Pharmacy, Phytochemical Research Laboratory, Federal University of Santa Maria (UFSM), 97105-900 Santa Maria, RS, Brazil

## Abstract

*Background. Duguetia furfuracea* is popular plant used in popular medicine.* Hypothesis/Purpose.* This claim evaluated the phytochemical composition of the hydroethanolic extract (HEDF), fractions of* Duguetia furfuracea*, and antioxidant and antifungal activity.* Methods.* The chemical profile was carried out by HPLC-DAD. The total phenolic contents and flavonoid components were determined by Folin-Ciocalteu and aluminium chloride reaction. The antioxidant activity was measured by scavenging of 2,2-diphenyl-1-picrylhydrazyl (DPPH) free radical and ferric reducing ability of plasma (FRAP) methods. The antifungal activity was determined by microdilution assay. Results. HPLC analysis revealed caffeic acid and rutin as major compounds (HEDF), caffeic acid and quercitrin (Mt-OH fraction), and quercitrin and isoquercitrin (Ac-OEt fraction). The highest levels of phenols and total flavonoids were found for Ac-OEt fraction, and the crude extract showed higher* in vitro* antioxidant potential. The antifungal activity showed synergic effect with fluconazole and EHDF against* C. krusei*, fluconazole and Mt-OH against* C. krusei* and* C. tropicalis*, and Ac-OE and fluconazole against* C. albicans*.* Conclusion.* The highest levels of phenols and total flavonoids were marked with antioxidant effect. This is the first report of bioactivity of the synergic effect of HEDF and fractions. More studies would be required to better clarify its mechanism of synergic action.

## 1. Introduction

The species* Duguetia furfuracea* is a shrub which belongs to the Annonaceae family, being popularly known as “araticum do cerrado,” “ata brava.” The leaves are popularly used for treatment of rheumatism and renal colic, antihyperlipidemic agent, and anorexic agent [1].

Studies with different structures of* D. furfuracea* have pointed out its cytotoxic, bactericidal, and antitumoral properties. Extracts from leaves and roots of* Duguetia furfuracea* presented larvicide activity against* Aedes aegypti* [2]. Alkaloids from peels of subterranean stem showed antitumor, trypanocide [3], and leishmanicidal activities [4, 5]. Extracts from subterranean parts of the plant displayed toxic effect to* Artemia salina* [4]. Our group demonstrated recently the prooxidant and insecticidal activity of the hydroalcoholic extract from leaves of* D. furfuracea* in* Drosophila melanogaster* [6]. Studies regarding antifungal and antioxidant properties of* Duguetia furfuracea* are scarce; thus the present work contributes to amplifying the knowledge about this species.

The antioxidant potential of the plants has been associated mainly with the presence of phenolic compounds. The chemical structure and reductive properties of these compounds make them active molecules in the scavenging of free radicals and in chelation of transition metals [7]. Secondary metabolites are substances with a generally complex structure playing several roles in the adaptation of plants to the environment; these substances have been attracting interest for their pharmacological and biotechnological properties such as antioxidant [8], anti-inflammatory [9], antimutagenic, anticarcinogenic, gastroprotection [10–12], and antimicrobial [13] ones.

The occurrence of fungal infections is increasing at alarming rates, especially among immunocompromised subjects, such as AIDS patients, transplanted patients, and neonates [14]. Among the pathogens, species of* Candida* are generally associated with these infections, whose incidence is attributed to a variety of factors, either exogenous or endogenous. More than 100 species of* Candida* are known and the frequency of distribution for* Candida spp.* varies in accordance with geographical location [15, 16].

Actually, conventional treatments for fungal infections are not fully effective, since the available drugs lead to secondary effects or to development of resistance [17]. Therefore, the search for new drugs and alternative therapies (including natural products) for the treatment of* Candida* infections has become critical. In this aspect, plants and their derivatives have been contributing to pharmacological research due to their potential as a source for a variety of biologically active ingredients used in drug development. The antimicrobial activity of plants has been identified in some species; however, it should be taken into consideration that besides its beneficial effects, the use of plants may interfere with conventional treatments by interaction with drugs, thus potentiating or minimizing clinical efficacy [18].

Our aim of the present study was to describe the phytochemical characterization,* in vitro* antioxidant potential and to evaluate, for the first time, the antifungal and/or modulatory activity of the hydroalcoholic extract, methanolic and ethyl acetate fractions of* Duguetia furfuracea*.

## 2. Materials and Methods

### 2.1. Drugs, Reagents, and Equipment

Sabouraud Dextrose Agar (semisolid) and Sabouraud Dextrose Broth were from Difco Laboratories (Michigan, MI, USA). Dimethyl sulfoxide (DMSO), acetonitrile, and formic, gallic, chlorogenic, ellagic, and caffeic acids were purchased from Merck (Darmstadt, Germany). Antifungal agents fluconazole and nistatina, catechin, quercetin, quercitrin, isoquercitrin, rutin, kaempferol, ethanol, methyl alcohol, hexane, ethyl acetate, Folin-Ciocalteu Reagent, gallic acid, sodium carbonate, aluminium chloride, quercetin, DPPH, ascorbic acid, sodium acetate, TPTZ, ferric chloride, and ferrous sulfate were purchased from Sigma Chemical Co. (St. Louis, MO, USA). High performance liquid chromatography (HPLC-DAD) was performed with the system of HLPC (Shimadzu, Kyoto, Japan), autosampler prominence (SIL-20A), equipped with high-pressure plunger pumps LC-20AT Shimadzu connected to DGU degasser 20A5 with integrator CBM 20A, UV-VIS detector DAD (diode) SPD-M20A, and software solution LC 1,22 SP1. The absorbance measurements were obtained using EnSpire® multimode plate reader (PerkinElmer, USA). All chemical products were of the highest analytical grade.

### 2.2. Collection of Plant Material

Leaves of* D. furfuracea* were collected from Barreiro Grande, Crato, Ceará (7°22′2.8′′S, 39°28′42.4′′W and altitude of 892 m above sea level), Brazil, in September 2011 and identified by Dr. Maria Arlene Pessoa da Silva. A voucher specimen (n. 6703) was deposited in the Herbarium Caririense Dárdano de Andrade Lima (HCDAL) of the Regional University of Cariri (URCA).

### 2.3. Preparation of the Hydroalcoholic Extract, Methanolic and Acetate Fractions

Leaves (1.050 g) of* D. furfuracea* were washed in running water, crushed, and put into glass flasks containing hydroalcoholic solution of extraction (99,8% of ethanol in distilled water) in the proportion of 1 : 1, for three days. The suspension was filtered, solvent evaporated under reduced pressure, and lyophilized to obtain 261.13 g of crude ethanolic extract. 80 g of this was partitioned with ethyl acetate and methanol to obtain g of 2.28 ethyl acetate fraction (EAF) and 75.6 g of methanolic fraction [19]. The procedure yielded 24.87% for HEDF, 94.5% for methanolic fraction (Mt-OH), and 3.35% for the ethyl acetate (Ac-OEt) fraction.

### 2.4. Identification and Quantitation of Phenolic Compounds of HEDF by HPLC

Mt-OH and Ac-OEt fractions were submitted to the chromatographic analysis of reversal phase. The chemical composition of the HEDF was previously determined by our group [6] using the same procedure described in this section.

The chromatographic analyses were performed under the same gradient conditions using the column C18 (4.6 mm × 250 mm) charged with particles of 5 *μ*m of diameter. The mobile phase was water containing 1% of formic acid (A), acetonitrile (B) and the composition gradient was 13% of B until 10 minutes and changed to obtain 20%, 30%, 50%, 60%, 70%, 20%, and 10% of B at 20, 30, 40, 50, 60, 70, and 80 minutes, respectively, according to Boligon et al. [20] with some modifications. Fractions were analyzed at a concentration of 5 mg/mL. The presence of ten antioxidant compounds was investigated: gallic acid, chlorogenic acid, ellagic acid, caffeic acid, catechin, quercetin, quercitrin, isoquercitrin, rutin, and kaempferol. The identification of these compounds was performed by comparing their retention time and the UV radiation absorption spectral to the commercial standards. The flow rate was 0.7 mL/min, injection volume 40 *μ*L, and the wavelength 254 nm to the gallic acid, 280 nm to the catechin, 325 nm to caffeic acid, ellagic acid, and chlorogenic acid, and 365 nm to quercetin, quercitrin, isoquercitrin, rutin, and kaempferol. All the samples and the mobile phase were filtered through a membrane filter of 0.45 *μ*m (Millipore) and, after this, they were degassed with ultrasonic bath before the use. The solutions of the standards of reference were prepared in a mobile phase of HPLC in a range of concentrations from 0.030 to 0.250 mg/mL to kaempferol, quercetin, quercitrin, isoquercitrin, rutin, and catechin and from 0.030 to 0.250 mg/mL to gallic acid, caffeic acid, ellagic acid, and chlorogenic acid. The peaks of chromatography were confirmed by comparing their retention time to the standards of reference and by the spectral of DAD (200 a 400 nm). All the operations of chromatography were performed at room temperature and in triplicate. The limit of detection (LOD) and the limit of quantification (LOQ) were calculated on the basis of the standard deviation of the responses and of the inclination of three analytical independent curves. LOD and LOQ were calculated as 3.3 and 10 *σ*/*S*, respectively, where *σ* is the standard deviation of the response and *S* is the slope of the calibration curve [20].

### 2.5. Determination of Total Phenols

The quantification of phenolic compounds was performed using the Folin-Ciocalteu method that involves the reduction of the reagent by phenolic compounds present in the samples forming a blue complex whose intensity increases linearly at 760 nm, as described by Swain and Hillis (1959) [21]. For the assays, 4 *μ*L of samples (HEDF, Mt-OH, and Ac-OEt) at a concentration of 100 *μ*g/mL to an incubation medium consisting of 0.1 N Folin-Ciocalteu reagent and 1.25% (w/v) Na_2_CO_3_ in a final volume of 284 *μ*L. After two hours of incubation in the dark, at room temperature, the absorbance was measured at 760 nm. The experiments were carried out in triplicate. The index of total phenolic compounds was expressed as equivalents of gallic acid* per* gram of the sample (mg GAEq/g), calculated through a curve of gallic acid, built with concentrations ranging from 50 to 500 *μ*g/mL.

### 2.6. Determination of Total Flavonoids

The quantification of flavonoids was made according to Quettier-Deleu et al. 2000 [22]. The method is based on the measurement of absorbance, at 415 nm, of the complex formed between flavonoid compounds and aluminum cation in ethanol. For the assays, samples were incubated with AlCl_3_ (2%) in a 1 : 1 reaction. The final volume of the reaction medium was 300 *μ*L. The experiments were carried out in triplicate. The index of total flavonoids was expressed as equivalents of quercetin* per* gram of the sample (mg QEq/g), calculated through a curve of quercetin, built with concentrations ranging from 0.625 to 25 *μ*g/mL.

### 2.7. Antioxidant Activity

#### 2.7.1. Scavenging Activity of the DPPH Radical

The antioxidant activity of the extract and fractions was also checked by the DPPH method as described by Brand-Williams et al. (1995) elsewhere [23] with minor changes. This test is based on the reduction of the stable free radical DPPH which presents a deep violet color in solution and turns to a yellowish color when neutralized. The mixture of the reaction was composed of 50 *μ*L of samples (extract and fractions), 50 *μ*L of solvent, and 100 *μ*L of solution and 0.3 mM of the radical DPPH in ethanol. The measurement of the absorbance was at 517 nm after 30 minutes. The samples (HEDF, Mt-OH, and Ac-OEt) were diluted in ethanol and water (1 : 1) and the standard substance, ascorbic acid, was diluted in water. All tests were made in triplicate. Results were expressed as IC_50_ values defined as the concentration of antioxidant required to sequester 50% of the DPPH radicals. IC_50_ was calculated by nonlinear regression.

#### 2.7.2. FRAP (Ferric Reducing Antioxidant Power)

The FRAP assay was conducted as described previously by Benzie and Strain (1996) [24]. FRAP solution consisted of 10 mM TPTZ, 20 mM ferric chloride in acetate buffer 0.3. In 96-well microtiter plate, we added 9 *μ*L of the samples (HEDF, Mt-OH, and Ac-OEt); 27 *μ*L of water; and 270 *μ*L of the FRAP solution. After incubation at 37°C for 30 minutes absorbance was taken at 595 nm. Samples readings were compared to a ferrous sulfate II standard curve. Results were expressed as *μ*M ferrous sulfate II (FeSO_4_) equivalents* per* gram of sample. Experiments were done in triplicate.

### 2.8. Antifungal Activity

#### 2.8.1. Culture Medium and Inoculums

The antifungal activity was evaluated using the standard fungal strains* C. albicans* (ATCC 40277),* C. krusei* (ATCC 6438), and* C. tropicalis* (ATCC 40042) donated by the Universidade Estadual da Paraíba. In the biological tests, we used the following culture medium: Sabouraud Dextrose Agar (semisolid) and Sabouraud Dextrose Broth (liquid) prepared according to the manufacturer's specifications. Fungal cultures kept at 4°C were transported to the Sabouraud Dextrose Agar medium and incubated at 35°C for 24 hours. As to the preparation of the inoculum, the pricked out strains were transferred to the sterile saline solution (0.9% NaCl), composing of a fungal suspension (inoculum) until obtaining the concentration of 10^5^ UFC/mL according to the scale of McFarland [25].

#### 2.8.2. Minimum Inhibitory Concentration Test: CIM and the Modulation of Standard Antifungal Action

The method of microdilution in sauce was used to determine the minimum inhibitory concentration (CIM). The samples (HEDF, Mt-OH, and Ac-OEt) were weighed and solubilized initially in dimethyl sulfoxide (DMSO) and diluted at 1024 *μ*g/mL using sterile distilled water (test solution).

We distributed 100 *μ*L of inocula, prepared previously, in each cavity of a 96-well microtiter plate and, thereafter, it was submitted to a serial double dilution using 100 *μ*L of the samples with concentrations that range from 1024 to 0.5 *μ*g/mL. The plates were transported to the incubator for 24 hours at 35°C [26]. The identification of CIM was performed through the visual observation of the turbidity provoked by the fungal growth, with the CIM being defined as the lowest concentration of the sample in which no fungal growth was observed [25].

To observe how these samples could affect the action of the standard antifungal agents against the strains tested, we used the method proposed by [27]. The extract and the fractions were tested using a subinhibitory concentration (MIC/8 = 64 *μ*g/mL). We distributed, in each well, 100 *μ*L of solution containing 1.675 *μ*L of liquid medium (Sabouraud Dextrose Broth) 10%; 200 *μ*L of inoculum (fungal suspension); and 125 *μ*L of the natural product (extract and fractions). After that, 100 *μ*L of the antifungal agents was added to the first cavity and following the serial dilution along the other cavities. The concentrations of the antifungal agents ranged from 1024 to 0.5 *μ*g/mL. The tests were performed in triplicate.

### 2.9. Statistical Analysis

The results of the tests were done in triplicate and expressed as geometric mean [28]. Statistical differences between samples were tested by analysis of variance ANOVA followed by Tukey's or Dunnett's* post hoc* test when necessary. The differences were considered significant when *P* < 0.05.

## 3. Results and Discussion

### 3.1. Identification and Quantification of Phenolic Compounds by HPLC

The chromatographic and spectral profile of Mt-OH and Ac-OEt fractions revealed the presence of gallic acid (*t*_*R*_ = 9.95 min; peak 1), catechin (*t*_*R*_ = 16.08 min; peak 2), chlorogenic acid (*t*_*R*_ = 20.14 min; peak 3), caffeic acid (*t*_*R*_ = 24.63 min; peak 4), ellagic acid (*t*_*R*_ = 37.29 min; peak 5); rutin (*t*_*R*_ = 39.87 min; peak 6); isoquercitrin (*t*_*R*_ = 44.93 min; peak 7); quercitrin (*t*_*R*_ = 48.15 min; peak 8); quercetin (*t*_*R*_ = 51.07 min; peak 9); and kaempferol (*t*_*R*_ = 61.56 min; peak 10).

**Figure 1:**
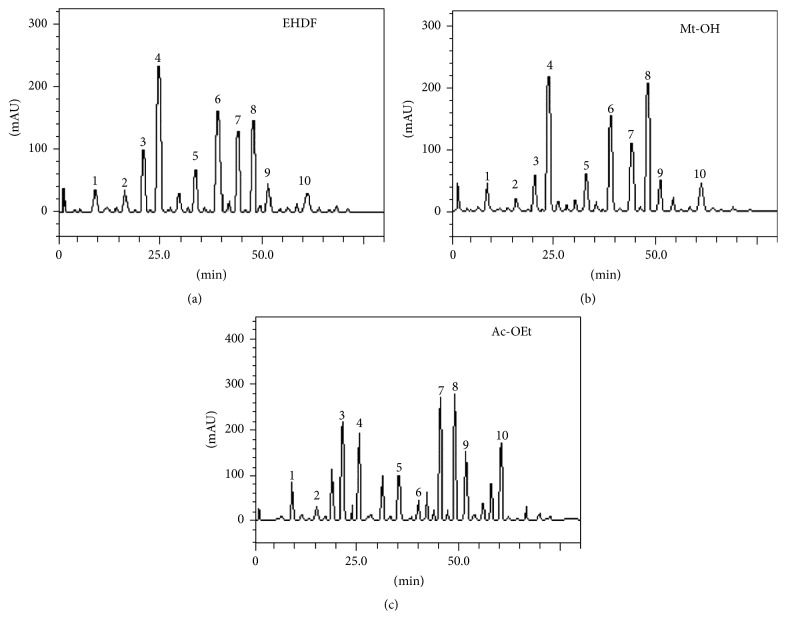
Elution profiles of high performance liquid chromatography analysis: (a) HEDF (modified from [6]), (b) methanolic fraction (Mt-OH) and (c) ethyl acetate (Ac-OEt). Gallic acid (peak 1), catechin (peak 2), chlorogenic acid (peak 3), caffeic acid (peak 4), ellagic acid (peak 5), rutin (peak 6), isoquercitrin (peak 7), quercitrin (peak 8), quercetin (peak 9), and kaempferol (peak 10). Calibration curve of the gallic acid: *Y* = 14286*x* + 1395.8 (*r* = 0.9996); catechin: *Y* = 15097*x* + 1189.3 (*r* = 0.9997); caffeic acid: *Y* = 12758*x* + 1259.7 (*r* = 0.9996); chlorogenic acid: *Y* = 13461*x* + 1275.3 (*r* = 0.9992); ellagic acid: *Y* = 13576*x* + 1346.4 (*r* = 0.9999); rutin: *Y* = 12845 + 1305.7 (*r* = 0.9999); quercetin: *Y* = 13560*x* + 1192.6 (*r* = 0.9991); isoquercitrin: *Y* = 12873*x* + 1325.6 (*r* = 0.9998); quercitrin: *Y* = 11870*x* + 1329.8 (*r* = 0.9993); and kaempferol: *Y* = 14253*x* + 1238.9 (*r* = 0.9997).

The main compounds present in the Mt-OH fraction were caffeic acid (32.47 ± 0.03 mg/g) and quercitrin (31.96 ± 0.03 mg/g) while catechin (2.69 ± 0.01 mg/g) and the gallic acid (5.47 ± 0.03 mg/g) were the least abundant. In the Ac-OEt fraction, major compounds were quercitrin (32.97 ± 0.037 mg/g) and isoquercitrin (31.56 ± 0.01 mg/g) while catechin (3.16 ± 0.02 mg/g) and rutin (5.49 ± 0.02 mg/g) were the least present (Figure 1 and Table 1). The chromatographic profile of HEDF demonstrated the presence of caffeic acid and rutin as major compounds (33.17 ± 0.03 mg/g and 20.05 ± 0.01 mg/g, resp.) while gallic acid (5.29 ± 0.01 mg/g) and catechin (5.31 ± 0.01 mg/g) were the least abundant [6].

The determination of total phenols and flavonoids is showed in Table 2. The content of total phenols and flavonoids was higher in Ac-OEt fraction (657.05 mg/EAG/g and 120.9 mg EQ/g, resp.), followed by Mt-OH fraction (289.33 mg/EAG/g and 76.26 mg EQ/g, resp.) and HEDF (231.26 mg EAG/g and 87.57 mg EQ/g, resp.). It is recognized that flavonoids are preferably extracted by ethyl acetate solvent.

The* in vitro* antioxidant potential of crude extract (HEDF) and fractions of* D. furfuracea* was evaluated by two different methods, FRAP, which measures the ferric reducing antioxidant power of compounds, and ability of sequestering the synthetic radical DPPH. The crude extract (HEDF) presented the best ferric reducing potential (166.73 ± 5.13 *μ*M of Fe^+2^/g of sample), followed by Mt-OH (126.43 ± 4.98 *μ*M of Fe^+2^/g of sample) and Ac-OEt fractions (118.20 ± 1.08 *μ*M of Fe^+2^/g of sample) (Table 3). The potential of scavenging of radical DPPH was expressed as IC_50_ in *μ*g/mL of extract or fractions and compared with the positive control ascorbic acid. HEDF presented the better antioxidant activity in the DPPH test with IC_50_ values of 33.15 *μ*g/mL when compared to Ac-OEt (39.32 *μ*g/mL) and Mt-OH (42.32 *μ*g/mL).

**Figure 2:**
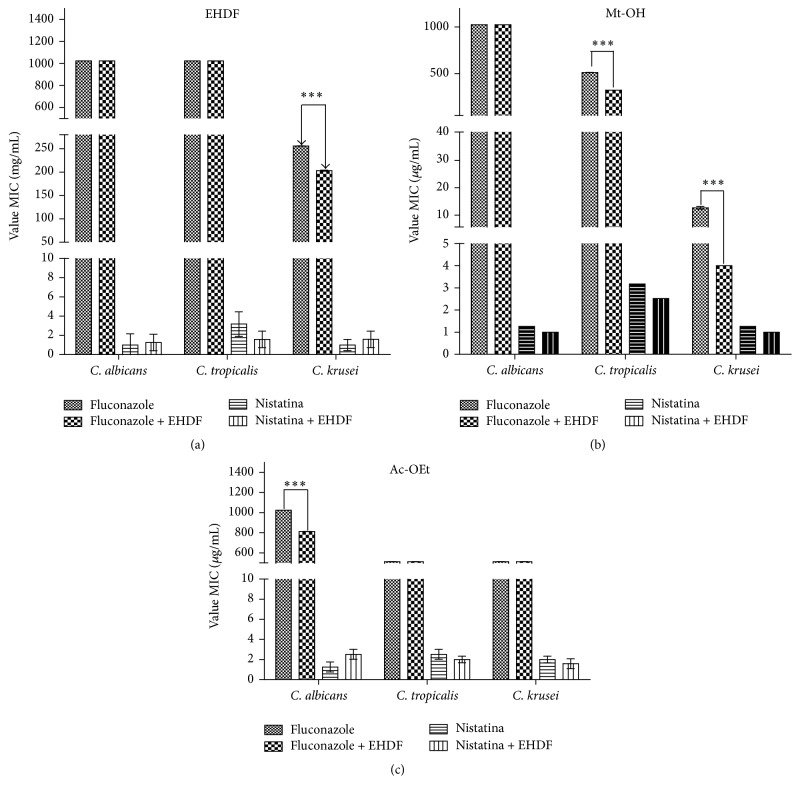
Modulatory activity of (a) HEDF (hydroalcoholic extract of* D. furfuracea*), Mt-OH, and Ac-OET* against the fungi C. albicans*,* C. krusei*, and* C. tropicalis* (concentrations ranging from 1024 *μ*g/mL to 0.5 *μ*g/mL). *P* < 0.001 related to control group; (b) Mt-OH (methanolic extract of* D. furfuracea*) against the fungi* C. albicans*,* C. krusei*, and* C. tropicalis* (concentrations ranging from 1024 *μ*g/mL to 0.5 *μ*g/mL); (c) Ac-OEt (ethyl acetate extract of* D. furfuracea*) against the fungi* C. albicans*,* C. krusei*, and* C. tropicalis* (concentrations ranging from 1024 *μ*g/mL to 0.5 *μ*g/mL). Statistical analysis: one-way ANOVA followed by Student-Newman-Keuls test. ^*∗∗∗*^ *P* < 0.001 versus fluconazole.

In this study, the antifungal potential of the hydroalcoholic extract of* D. furfuracea* (HEDF) and methanolic (Mt-OH) and ethyl acetate (Ac-OEt) fractions was tested against standard strains of* C. albicans*,* C. tropicalis*, and* C. krusei*. According to the results, both extract and fractions presented minimal inhibitory concentration (CIM) ≥1024 *μ*g/mL against all the fungi strains tested. However, the extract and the fractions of* D. furfuracea* presented synergic effect when they were associated with fluconazole, indicating its modulatory action against fungi when associated with clinically relevant drugs. The HEDF and Mt-OH fraction potentialized the effect of the fluconazole when tested against* C. krusei* as observed in Figures 2(a) and 2(b). The methanolic fraction also presented synergism with fluconazole against* C. tropicalis* (Figure 2(b)) and Ac-OEt fraction potentialized the effect of fluconazole against* C. albicans* (Figure 2(c)).

## 4. Discussion

Phenolic compounds and some of their derivatives are known by their antioxidant properties. The antioxidant activity of some medicinal plants is correlated to the total phenolic and flavonoids indexes [29]. The level of total phenols for* D. furfuracea* extract and fractions is comparable with other Brazilian medicinal plants of* Duguetia* genus [30, 31]. Although the total index of phenols and flavonoids has been higher in the fractions than in crude extract, it is possible to connect these compounds with* in vitro* antioxidant activity, as determined by FRAP and DPPH methods. Though the* in vitro* antioxidant activity was higher in the crude extract than the fractions, this analysis suggests that other compounds in the crude extract of* D. furfuracea* contribute to its more effective antioxidant activity [32]. In a previously published study by our group, the crude extract (HEDF) was demonstrated to present alkaloids in its phytochemical constitution [6]. In this work we can see a direct correlation between the concentration of flavonoids and antioxidant FRAP activity (*r* = −0.801). Those results were confirmed with others results present in literature [33–35].

The primary mechanism of fluconazole's action occurs by the inhibition of the fungal enzyme lanosterol 14*α*-demethylase (CYP51), which is a cytochrome enzyme P-450, involved in the synthesis of the ergosterol, the most important sterol in the fungal cell membrane [36]. It is known that many medicinal plants may modulate the activity of several antimicrobial agents [37, 38]. In a previous study, the aqueous extract of the leaves and fractions of* D. furfuracea* when combined with aminoglycosides presented synergic activity against* Escherichia coli* and* Staphylococcus aureus* [39].

Phenolic compounds and flavonoids have demonstrated potential therapeutic activities as antifungal, antibacterial, and antioxidant agents [40, 41]. Although the mechanisms underlying antimicrobial pharmacology of the phenolic compounds are rather variable, many of them act by promoting damage to the function of the cell membrane or cell wall [42]. The analysis by HPLC of the extract and fractions of* D. furfuracea*, as described previously, revealed the predominance of the following compounds: caffeic acid, chlorogenic acid, rutin, quercitrin, and isoquercitrin. There is a study that demonstrated that chlorogenic acid presented antifungal activity against the yeast of the gender* Candida* [43]. Six flavonoids that were isolated from plants, among them, rutin, presented antibacterial and antifungal activity [40].

Sun et al. (2004) showed the influence of phenolic compounds in fluconazole antifungal properties. This paper shows that the concentration of fluconazole in* C. albicans* was found to be increased with the increment of the phenolic compounds concentration when they were in combination. This result corroborated synergetic activity present in this work [44].

It is possible to speculate that some of these chemical constituents, especially the flavonoids, are responsible for the pharmacological properties found. However, the isolation and the activity of alkaloids and acetogenines have stood out in studies with plants of the Annonaceae family. The biological activity as antimicrobial capacity and antioxidant activity present in* A. muricata* can be attributed to the presence of acetogenines [37, 45]. Alkaloids as aphorphinoids present the bark of* Annona salzmannii* D. C. was responsible for the antioxidant and antimicrobial capacity [46].

## 5. Conclusion

We can conclude that the crude extract of* D. furfuracea* (HEDF) and its methanolic (Mt-OH) and ethyl acetate (Ac-OEt) fractions have an important antioxidant activity (*in vitro*) when compared to other natural compounds. The crude extract (HEDF) presented highest antioxidant activity* in vitro* when compared to Mt-OH and Ac-OEt, as determined by DPPH and FRAP methods. However, it was not possible to observe a positive correlation between the antioxidant activity and the total index of phenols and flavonoids identified, indicating that compounds other than phenolics may contribute to the antioxidant potential of the plant extracts. The crude extract and fractions of* D. furfuracea* presented a synergistic activity with fluconazole when tested against strains of* C. albicans, C. tropicalis, and C. krusei*, indicating a potential antifungal activity via modulation of clinically used drugs against fungal infections. More studies are needed to clarify the mechanisms involved in this phenomenon as well as other potential biomedical and biotechnological applications of* D. furfuracea*.

## Supplementary Material

Duguetia furfuracea belonging to the Annonacea family is popularly known as “ata brava” being widely used in popular medicine. The chemical profile of hydroethanolic extract and fractions was carried by HPLC–DAD and revealed caffeic acid and rutin as major compounds. The antifungal activity was showed significate synergic effect with fluconazole. Studies regarding antifungal and antioxidant properties of *Duguetia furfuracea* are scarce, thus the present work contributes to amplify the knowledge about this species.

## Figures and Tables

**Table 1:** Quantification of phenolic compounds of HEDF. Me-OH and Ac-OEt.

Compounds	HEDF (modified from [6])	Me-OH fraction	Ac-OEt fraction	LOD	LOQ
	mg/g	mg/g	mg/g	*μ*g/mL	*μ*g/mL
Gallic acid	5.29 ± 0.01	5.47 ± 0.03	9.85 ± 0.01	0.015	0.049
Catechin	5.31 ± 0.01	2.69 ± 0.01	3.16 ± 0.02	0.032	0.105
Chlorogenic acid	16.03 ± 0.02	7.18 ± 0.01	25.78 ± 0.01	0.009	0.029
Caffeic acid	33.17 ± 0.03	32.47 ± 0.03	21.90 ± 0.03	0.024	0.078
Ellagic acid	7.30 ± 0.01	7.25 ± 0.01	11.17 ± 0.01	0.013	0.042
Rutin	20.05 ± 0.01	19.67 ± 0.02	5.49 ± 0.02	0.027	0.090
Isoquercitrin	18.61 ± 0.01	14.83 ± 0.01	31.56 ± 0.01	0.008	0.026
Quercitrin	19.07 ± 0.02	31.96 ± 0.03	32.97 ± 0.037	0.035	0.114
Quercetin	5.87 ± 0.01	6.95 ± 0.01	18.73 ± 0.01	0.019	0.063
Kaempferol	5.36 ± 0.01	6.91 ± 0.02	20.98 ± 0.02	0.026	0.085

Results are expressed as mean ± SE of three determinations. LOD: limit of detection. LOQ: limit of quantification.

**Table 2:** Total phenolic contents and flavonoids present in extract and fractions of *D. furfuracea*.

Samples	Total phenolic contents	Total flavonoids
	mg of GEA/g of the sample	mg of EQ/g of the sample
HEDF	231.26 ± 1.15^a^	76.26 ± 2.73^a^
Mt-OH	289.33 ± 1.22^b^	87.57 ± 2.48^b^
Ac-OEt	657.05 ± 6.33^c^	120.9 ± 2.53^c^

The values were expressed as mean ± SD (*n* = 3); EAC: equivalent of gallic acid; EQ: equivalent of quercetin. Averages followed by different letters differ by Tukey's test at *P* < 0.05.

**Table 3:** Antioxidant activity of HEDF and fractions of *D. furfuracea*.

Samples	Sequestering of the radical DPPH	FRAP
	EC_50_ (*μ*g/mL)	*μ*M Fe^+2^/g of the sample
HEDF	33.15^b^	166.73 ± 5.13^a^
Mt-OH	42.32^c^	126.43 ± 4.98^b^
Ac-OEt	39.32^c^	118.20 ± 1.08^b^
Asc. Ac.	17.50^a^	—

The values were expressed as mean ± SD (*n* = 3); EFe^+2^ = equivalent of iron; HEDF (hydroalcoholic extract of *D. furfuracea*); Mt-OH (methanolic fraction); Ac-OEt (ethyl acetate fraction); and Asc. Ac. (ascorbic acid). Results are expressed as mean ± SEM (*n* = 3). Averages followed by different letters differ by Tukey's test at *P* < 0.05.

## Abbreviations

HEDF: Hydroethanolic extract of* Duguetia furfuracea*
Mt-OH: Methanolic fractions of* Duguetia furfuracea*
Ac-OEt: Acetate fractions of* Duguetia furfuracea*
HPLC DAD: High performance liquid chromatography with diode array
DPPH: 2,2-Diphenyl-1-picrylhydrazyl
FRAP: Ferric reducing antioxidant power.
