# Supplementary material for: Phytochemical Composition, Antifungal and Antioxidant Activity of Duguetia furfuracea A. St.-Hill
Source: Oxid Med Cell Longev. 2016 Apr 5;2016:7821051. doi: 10.1155/2016/7821051 (PMC4835657; doi:10.1155/2016/7821051)
Supplement: Supplementary file 1 — Duguetia furfuracea belonging to the Annonacea family is popularly known as “ata brava” being widely used in popular medicine. The chemical profile of hydroethanolic extract and fractions was carried by HPLC–DAD and revealed caffeic acid and rutin as major compounds. The antifungal activity was showed significate synergic effect with fluconazole. Studies regarding antifungal and antioxidant properties of Duguetia furfuracea are scarce, thus the present work contributes to amplify the knowledge about this species. [file 7821051.f1.docx]

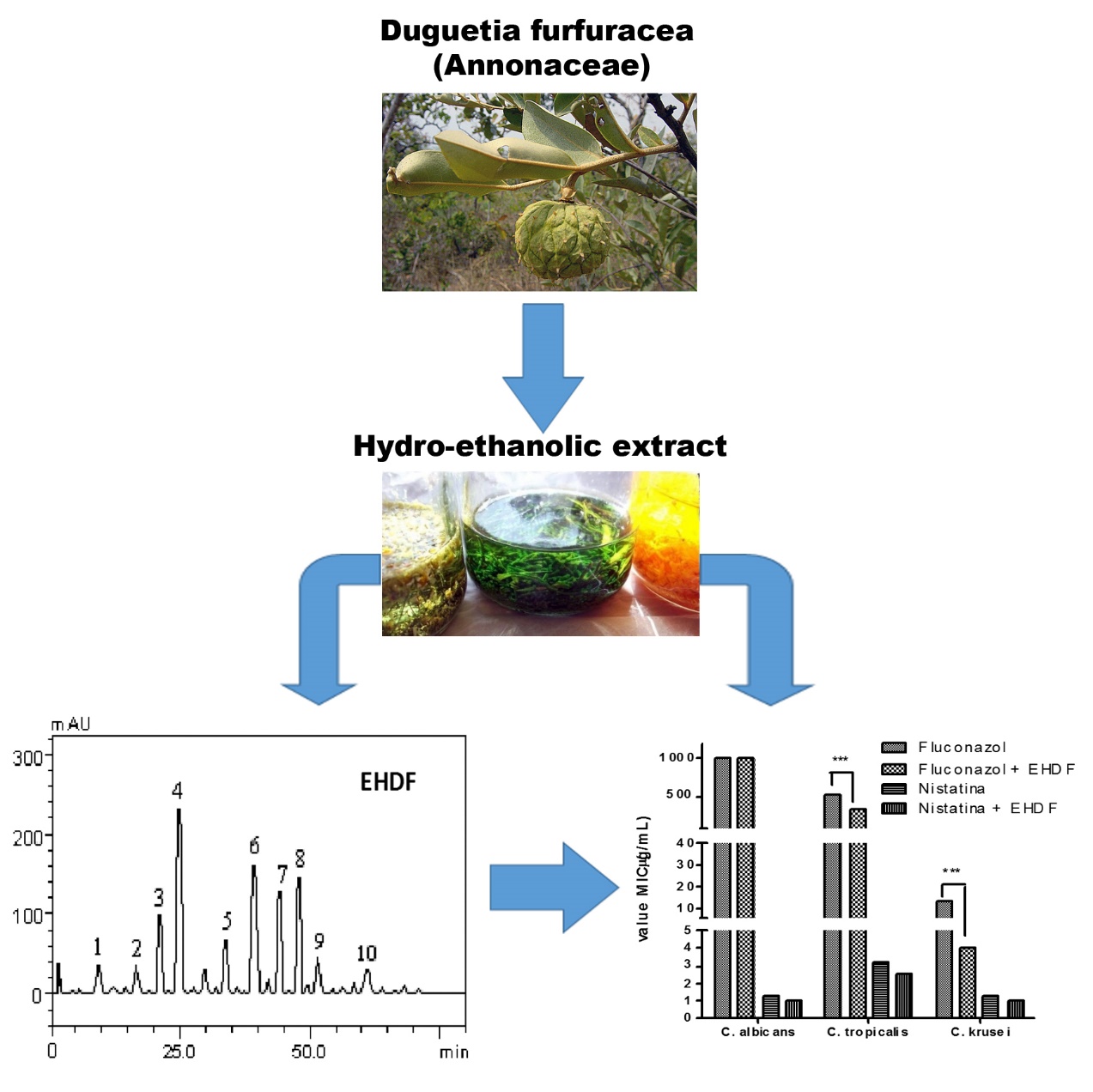


Duguetia furfuracea belonging to the Annonacea family is popularly known as “ata brava” being widely used in popular medicine. The chemical profile of hydroethanolic extract and fractions was carried by HPLC–DAD and revealed caffeic acid and rutin as major compounds. The antifungal activity was showed significate synergic effect with fluconazole. Studies regarding antifungal and antioxidant properties of *Duguetia furfuracea* are scarce, thus the present work contributes to amplify the knowledge about this species.
